# Anti-Inflammatory, Antithrombotic, and Antioxidant Properties of Amphiphilic Lipid Bioactives from Shrimp

**DOI:** 10.3390/ph18010025

**Published:** 2024-12-28

**Authors:** Alexandros Tsoupras, Paschalis Cholidis, Dimitrios Kranas, Evangelia Aikaterini Galouni, Anna Ofrydopoulou, Pavlos Efthymiopoulos, Katie Shiels, Sushanta Kumar Saha, George Z. Kyzas, Chryssa Anastasiadou

**Affiliations:** 1 Hephaestus, Laboratory, School of Chemistry, Faculty of Sciences, Democritus University of Thrace, St Lukas, 65404 Kavala, Greece; paoholi@chem.duth.gr (P.C.); dikrana@chem.duth.gr (D.K.); evgalou@chem.ihu.gr (E.A.G.); anofrid@chem.ihu.gr (A.O.); pefthym@chem.duth.gr (P.E.); kyzas@chem.duth.gr (G.Z.K.); 2 Centre for Applied Bioscience Research, Technological University of the Shannon: Midlands Midwest, Moylish Park, V94 E8YF Limerick, Ireland; katie.shiels@tus.ie (K.S.); sushanta.saha@tus.ie (S.K.S.); 3 Fisheries Research Institute, Nea Peramos, 64007 Kavala, Greece

**Keywords:** shrimp, amphiphilic bioactives, marine phenolics, carotenoids, polar lipids, UFA, MUFA, PUFA, omega-3, anti-inflammatory

## Abstract

**Background/Objectives:** Marine organisms, including shrimps, have gained research interest due to containing an abundance of bioactive lipid molecules.This study evaluated the composition and the in vitro biological activities of amphiphilic bioactive compounds from four different wild shrimp species: *Litopenaeus vannamei*, *Penaeus kerathurus*, *Aristaeomorpha foliacea*, and *Parapenaeus longirostris*. **Methods:** Total lipid (TL) extracts were obtained from shrimp and separated into total amphiphilic (TAC) and total lipophilic (TLC) compounds. Phenolic (TPC) and carotenoid (TCC) contents, antioxidant activities (DPPH, ABTS, FRAP assays), and biological effects on platelet-activating factor (PAF) and ADP-induced platelet activation were evaluated. Structural analyses were performed using ATR-FTIR spectroscopy, while LC-MS was used to elucidate the fatty acid composition and overall structure of polar lipids (PLs) present in shrimp TAC extracts. **Results:** TAC extracts, rich in phenolics, carotenoids, PL, and unsaturated fatty acids (UFAs), exhibited stronger anti-inflammatory and antithrombotic activities compared with TLC extracts, which showed potent antioxidant capacity. Significant amounts of UFAs, such as the monounsaturated fatty acid (MUFA) oleic acid (C18:1n9) and omega-3 (n3) polyunsaturated fatty acids (PUFAs) like eicosapentaenoic acid (EPA; C20:5n3) and docosahexaenoic acid (DHA; C22:6n3), were detected in the PLs of shrimp TAC extracts, with favorable anti-inflammatory values for their n6/n3 PUFA ratio. Shrimp amphiphilic bioactives present in the TAC extracts provide anti-inflammatory effects against the PAF pathway and antithrombotic effects against ADP and eicosanoid pathways. **Conclusions**: The overall findings support further study on the use of shrimp extracts rich in anti-inflammatory, anti-thrombotic, and antioxidant amphiphilic bioactives as ingredients to produce new bio-functional health-promoting products, in the context of sustainable development and circular economy.

## 1. Introduction

During the past decade, marine bioactives have garnered significant interest due to their health–promoting impact coupled with their ample availability. With respect to sustainability, another advantage of marine organisms is that they can be farmed in aquaculture, a process of breeding, raising and harvesting fish, shellfish, and aquatic plants. This represents a significant advantage especially for crustaceans like shrimps, due to cost-effectiveness which enables mass production. Many studies have been conducted on marine lipids and their effects on several diseases such as cardiovascular diseases, skin issues, and neurological diseases [1,2]. More specifically, long-chain omega-3 (n3) polyunsaturated fatty acids (PUFAs) and polar lipids (PLs) bearing such n3 PUFAs within their structures have been proven to improve vascular health as well as to decrease inflammation, blood pressure, and oxidative stress [1]. Therefore, they have become an integral part of a healthy and nutritional diet, with experts suggesting that people should consume a specific quantity of seafood weekly [3].

The fact that shrimps can be harvested in abundant quantities through aquaculture gives a huge advantage to the industry, because high amounts of bioactive compounds are produced. However, to achieve maximum efficiency in these projects, it is important to evaluate factors that can affect the lipid content in these crustaceans. Some of these parameters are related to the sex of the shrimps, the season, and environmental conditions such as temperature, pH, water salinity, the geographical position of the ocean, and the depth that shrimp species are located, as well as potential diseases/pathogens that can affect shrimps, such as parasites and viruses [2]. Studies conducted on various parasites in different shrimp species, such as the branchial ectoparasite isopod *Probopyrus ringueleti* and hepatopancreatic microsporidiosis caused by *Enterocytozoon hepatopenaei* (EHP), induced the down-regulation of lipid metabolism and affected their energy [4,5]. Thus, it is important to find solutions to this matter, including preventive measures such as water quality testing and sanitation, as well as monitoring and early diagnosis. Overall, several regulations and procedures are required in order to establish the isolation and industrial utilisation of natural bioactives.

Nevertheless, so far, there has been insufficient research on shrimps regarding their bioactive molecules, lipid composition, and overall benefits. Shrimps are naturally rich in UFAs like monounsaturated fatty acids (MUFAs) and PUFAs, as well as several classes of PLs [6]. Particularly important UFAs found in shrimps include n3 PUFAs like docosahexaenoic acid (DHA, C22: 6n3) and eicosapentaenoic acid (EPA, C20: 5n3). Shrimp consumption can nourish the human body with the recommended levels of n3 PUFAs, which are associated with cardiovascular improvement. Such constituents are very important for our body, mainly because we are entirely unable to produce them without any previous seafood consumption. Subsequently, due to their high anti-inflammatory and antithrombotic activities, their structural role in membranes and their function as sources of various metabolites are linked to the treatment of many diseases [7].

Apart from FAs, shrimps also contain several other beneficial lipid bioactives that are believed to exert antioxidant and anti-inflammatory protective properties on the cardiovascular system. For example, in addition to bioactive PLs and n3 PUFAs, many crustaceans, including shrimps, are enriched with antioxidant and anti-inflammatory biomolecules such as marine carotenoids and phenolics [8]. The most important antioxidant commonly found in relatively high concentrations in such marine organisms is astaxanthin, which can be either free or esterified with lipids [9]. Astaxanthin (3,3′-dihydroxy-beta, beta-carotene-4,4′-dione) is a xanthophyll carotenoid with remarkably high antioxidant activity, more than any other carotenoid and even some vitamins. 

According to recent studies on the pathogenesis of various diseases, oxidative stress and chronic inflammation are leading causes in the occurrence of several chronic diseases. Oxidative stress is caused by an imbalance between reactive oxygen species (ROS) and natural antioxidants. Τhis imbalance causes damage to cell components by lipid peroxidation, protein oxidation, DNA damage, and the activation of signaling pathways related to inflammation, such as nuclear factor-kappa B (NF-κB) and mitogen-activated protein kinases (MAPKs), which cause the production of pro-inflammatory cytokines. Inflammation is characterised as a natural response of the immune system against several types of implication/injury or metabolic dysfunction. Oxidative stress and inflammation are closely related, and they act synergistically by producing increased amounts of pro-inflammatory mediators such as interleukins, tumor necrosis factor (TNF), platelet activating factor (PAF), eicosanoids, and many more. Additionally, the World Health Organisation (WHO) states that cardiovascular diseases are the leading cause of death globally, induced by inflammation and oxidative stress causing platelet aggregation, which is the main reason for the formation of thrombus in diseases such as atherosclerosis and venous thromboembolism. Even though platelet aggregation is closely linked to CVDs, it also contributes to neurovascular diseases like ischemic stroke by obstructing the blood flow, cancer by promoting metastasis, and inflammatory diseases, since platelet aggregation is linked to inflammation, such as in rheumatoid arthritis and systemic lupus erythematosus [10].

Research on natural antioxidant, anti-inflammatory, and anti-thrombotic bioactive products, especially of marine origin, is essential, due to the important properties that these bioactives have against oxidative stress, chronic inflammation, and clot formation [1,2]. Antioxidants scavenge and neutralise free radicals, which is crucial in the prevention of cellular damage, with subsequent health benefits against chronic diseases, including CVDs and several types of cancer [2,11]. Anti-inflammatory bioactives of marine origin can modulate inflammatory pathways by acting as inhibitors, which indicates the significant effects they have against health conditions related to inflammation, such as heart disease, diabetes, etc. [1,2,10]. Furthermore, anti-thrombotic agents can prevent formation of clots, reducing the risk of developing conditions like stroke or myocardial infraction. These properties indicate the contribution of anti-thrombotic agents to cardiovascular health [1,10]. Thus, the utilisation of bioactives from natural products and especially those of marine origin can be crucial in managing various health conditions.

Shrimp extracts have shown positive effects on other diseases as well, including diabetes, Alzheimer’s disease, obesity, cancer, neurological diseases, etc. [9,12]. For example, shrimp lipids seem to exhibit protective action by using restraining compounds to stop cancer cell division. Shrimps apparently offer a plethora of benefits that are yet to be fully explored, analysed, and experimented upon. However, little is known about amphiphilic compounds from shrimps. 

Thus, within the present study, the total amphiphilic compounds (TACs) and total lipophilic compounds (TLCs) of total lipid (TL) extracts from four different shrimp species, *Aristaeomorpha foliacea*, *Litopenaeus vannamei*, *Parapenaeus longirostris*, and *Penaeus Kerathurus*, which have different sizes, depth preferences, and several other characteristics, were assessed for their total phenolic (TPC) and carotenoid (TCC) contents and for their in vitro antioxidant activities, as well as for their anti-inflammatory and antithrombotic properties, in human platelets. Structural elucidation and evaluation of the active structural relationships of these shrimp bioactives were examined utilising both ATR-FTIR spectroscopy and LC-MS analysis.

## 2. Results and Discussion

### 2.1. Yield of Extraction

The extraction and separation of amphiphilic (TAC), lipophilic (TLC), and total lipid (TL) compounds from the four shrimp species was based on a modification of the Bligh and Dyer extraction method [13] and the counter-current distribution technique of Galanos and Kapoulas [14], as described by Tsoupras and others [15]. This combination of methods has been applied in previous studies to efficiently recover and separate amphiphilic compounds like PLs and lipophilic compounds like neutral lipids (NL) from various natural sources, including marine organisms, for further evaluation of their bioactive properties [1]. In fact, it has been reported that unlike other extraction methods, this combined technique ensures the preservation of the lipid components’ bioactivity during the extraction process, as verified by the high bioactivity that the tested extracts exhibited in the antioxidant and anti-inflammatory/anti-thrombotic activity assays.

Table 1 shows the extraction yields for all TAC, TLC, and TL extracts recovered from each of the samples from the four shrimp species assessed, expressed in g of dried extract per 100 g of shrimp, respectively. As indicated, higher amounts of amphiphilic than lipophilic lipid components were found in all shrimp. The above result is significant as it has been reported that unlike lipophilic and total lipids, which exhibit low and intermediate bioactivities, respectively, amphiphilic lipid bioactives like marine PLs are the most bioactive lipid class against inflammation, thrombosis, and associated disorders [1]. Also, by applying Tsoupras et al.’s method, which constitutes a modification of other lipid extraction techniques, satisfactory extraction yields were achieved, mainly of amphiphilic lipid components from shrimp. These were comparable to or even higher than other yields previously reported for the extraction of lipids from shrimp.

More specifically, within all shrimp species assessed, the red-colored benthopelagic giant shrimp, *Aristaeomorpha folicea*, which is a decapod crustacean that inhabits deep waters and was harvested from the Sea of Rhodes (southern eastern Aegean Sea, Greece), showed the highest yield of both TAC and TLC extracts in comparison to the other shrimp species, and thus, the highest TL yield too. The second highest yield of both TAC and TL extracts was observed in the deep-water rose shrimp, *Parapenaeus longirostris*, which was harvested from the Thracian Sea, from which the TAC extract yield was found to be approximately six times higher than the TLC extract yield. Unexpectedly, the Pacific white shrimp, *Litopenaeus vannamei*, and the brown-colored caramote prawn, *Penaeus kerathurus,* which was also harvested from the Thracian Sea, showed the lowest yield of TLs. Between these two shrimp species, *Penaeus kerathurus* showed higher TAC extract yield than *Litopenaeus vannamei*.

Moreover, within each shrimp species, three out of the four shrimp species, *L. vannamei*, *P. kerathurus*, and *P. longirostris*, showed higher TAC extract yields compared with their TLC extract yields (*p* < 0.05). There was a tendency towards a similar relationship between the TAC and TLC extract yields in *A. foliacea* too, but without statistical significance (*p* > 0.05). Nevertheless, even though the overall yield in TLs from all shrimp species assessed was much lower than that observed in oily fish species like salmon and herring, the TAC yields for all shrimp species were still found to be comparable to the TAC yields from other marine organisms [1] and from previous studies in shrimp [6]. Interestingly, unlike oily fish like salmon and herring, in which the TLC extract yields (NL) are usually much higher than the TAC (PL) extract yields [1], in three out of four shrimp species (*L. vannamei*, *P. kerathurus*, and *P. longirostris*), the yield of TAC was higher than the yield of TLC, which further supports the success of the applied extraction procedure that was focused on separating the TAC from these shrimp species. In contrast, only in the brown-colored caramote prawn *Penaeus kerathurus* was the TLC yield higher than the TAC yield. Overall, these results are in accordance with previous studies showing that shrimps are a rich marine source of extracts rich in amphiphilic compounds like PLs [16].

### 2.2. Total Carotenoid Content (TCC) of Extracts from All Shrimp Species

As shown in Table 2, in which the total carotenoid contents of TAC, TLC, and TL extracts from all shrimp species are presented expressed in mg of β-carotene equivalents (CE) per g of dried extract, fewer amphiphilic and more lipophilic carotenoids were present in the TLC extracts, which yielded carotenoids in a yield approximately one order of magnitude higher than that observed for TAC extracts in all shrimp species (*p* < 0.05). Much lower amounts of more amphiphilic (probably of smaller MW) carotenoids migrated into the TAC extracts during the counter-current separation process, further suggesting a successful separation of the more amphiphilic compounds from the more lipophilic ones. Independently of the different colors and sizes of these shrimp species, the carotenoid yields in all TLC extracts from all shrimp species were found to be similar, which was also observed when the carotenoid contents of all TL extracts were compared between all species, as well as when the carotenoid contents of all TAC extracts were compared between these species.

Indeed, the presence of lipid soluble antioxidant carotenoid bioactives like astaxanthin has been reported in lipid extracts from shrimp [9] and from other marine sources like salmon [1], probably due to their fish feed being rich in carotenoids, including fish feed derived from microalgae and small shrimps. Interestingly, within the present study, higher TCC yields were observed in the TL and TLC extracts for all four shrimp species assessed, in comparison to previous studies for these specific shrimp species [16,17,18]. The presence of such marine carotenoids in shrimp extracts further supports their antioxidant capacity and relevant health-promoting effects against oxidative stress and associated disorders [1]. 

### 2.3. Total Phenolic Content (TPC) in Extracts from All Shrimp Species

Table 3 shows the total phenolic contents in TAC, TLC and TL extracts from all shrimp species assessed, expressed in mg of gallic acid equivalent (GAE) per g of dried extract. As shown, the total marine phenolics were detected only in the TAC extracts from all shrimp species, reflecting the phenolic content of their TL extracts too, since phenolic compounds were not detected in the TLC extracts of any of the shrimp species assessed. 

Within all the assessed shrimp species, *L. vannamei* showed the highest TPC (*p* < 0.05 in comparison to all other shrimp species), followed by *P. kerathurus*, which showed the second highest TPC (*p* < 0.05) in comparison to all the other shrimp species. Interestingly, the results for all the shrimp species were found to be comparable to previous data, even though those were not expressed as GAE but in phloroglucinol equivalents [19]. The presence of such marine phenolics in all shrimp species assessed also seems to protect against the unfavorable effects of enzymatic browning in crustaceans [20]. They can also provide protection against oxidative stress and inflammation in humans who consume shrimps or shrimp extracts rich in phenolic bioactives, due to the important antioxidant and anti-inflammatory properties reported for such marine-derived bioactives [11].

### 2.4. Antioxidant Activity of TAC, TLC, and TL Extracts from Shrimps

The results of the antioxidant assay methods ABTS, DPPH, and FRAP for all the examined extracts are presented in Table 4. As shown in this table, for both the ABTS and DPPH assays, the most lipophilic TLC extracts of *L. vannamei*, *P. kerathurus*, and *P. longirostris* showed higher antioxidant activities, reflecting the higher carotenoids content in these TLC extracts from their TAC extracts, suggesting that marine carotenoids present in the TLCs exhibit higher antioxidant capacity than the low quantities of marine phenolics present in their TAC extracts. 

In contrast, the TAC extract from *A. foliacea* showed a stronger antioxidant effect than its TLC extract, seeming to reflect a synergistic effect of its phenolic content with some of its carotenoids (of lower Mr) that migrated to its TAC extracts were are co-present with its low phenolic content, since TAC extracts from this shrimp species showed higher carotenoid content compared with the TAC extracts from all the other shrimp species. 

Interestingly, different from the outcomes observed in the ABTS and DPPH assays, other outcomes were observed in the FRAP assay for the TAC and TLC extracts of these shrimp species. More specifically, the TAC extracts of *L. vannamei* and *P. kerathurus* showed similar antioxidant capacity to the TLC extracts for these shrimp species, while for *A. foliacea*, only the TAC extracts showed an antioxidant effect (its TLC extracts did not show such an effect in this assay). In contrast, for the shrimp species *P. longirostris*, only the TLC extracts showed an antioxidant effect (its TAC extracts did not show such an effect in this assay). It should be mentioned that the FRAP assay revealed no statistically significant difference in overall antioxidant capacity of the TL extracts between *P. longirostris*, *L. vannamei*, and *P. kerathurus*, while the TL extracts of *A. foliacea* showed the lowest overall antioxidant capacity. 

Both the ABTS and DPPH assays measured the free radical scavenging activities, meaning the relative abilities of antioxidants to scavenge the ABTS and DPPH radicals in the aqueous phase, respectively. The FRAP assay measured the antioxidant potential in samples through the reduction of ferric iron (Fe^3+^) to ferrous iron (Fe^2+^) by antioxidants present in the samples, meaning the test reflected the ferric-reducing ability of plasma, which is closer to in vivo conditions, as previously mentioned [15,21]. Thus, the higher activity observed in the FRAP assay for the TAC extracts of *L. vannamei*, *P. kerathurus*, and *A. foliacea* species, which were found to be similar (for *L. vannamei* and *P. kerathurus*) or even much higher (for *A. foliacea*) than their TLC activities in the same assay, further reflects the abilities of the TAC extracts to demonstrate antioxidant effects in conditions that are closer to in vivo situations, like in the FRAP assay (ferric-reducing ability of blood plasma) in comparison to the ABTS and DPPH assays, under morein vitro conditions, where the TAC showed lower antioxidant capacities. 

The antioxidant activity of the four investigated shrimp species, *L. vannamei*, *P. kerathurus*, *A. foliacea*, and *P. longirostris*, showed varying ranges according to the assay used and the targeted compounds. The DPPH-scavenging activity of the *L. vannamei* extract was similar to the values found in marine fungi and in shrimp extracts of *P. varians* [22,23], which showed strong antioxidant potential due to their high carotenoid content. Carotenoids have the ability to scavenge free radicals effectively by physical quenching (energy transfer) or chemical reactions (electron transfer, addition reactions), due to their molecular structure, which has a large number of conjugated double bonds [24,25]. The reported ABTS values for *L. vannamei* ranged from 8 to 14 mg TE/g, which was in close agreement with the values for *P. varians, P. serratus*, and *L. stylirostris* and higher than those for salmon species, which were approximately 1.61–1.73 μmol TE/g [23,26,27]. The FRAP values for *P. kerathurus* were in close agreement with values previously reported for shrimp shell waste from *P. serratus* (FRAP values of approximately 1773 TE/g DW). *A. foliacea* exhibited slightly higher antioxidant activity than the other shrimp species, comparable to brackish shrimp *P. serratus* and *P. varians* [23], demonstrating potential for antioxidant activity in deep-water shrimp species. Similar to *P. kerathurus* and *L.vannamei, P. longirostris* exhibited a pattern of reliable antioxidant activity with ABTS-scavenging values ranging from 7.4 to 66.2 μmol TE/g. These values were higher than those found in salmon species but marginally lower than those reported for *P. varians* [23,26]. These results highlight the value of shrimp’s antioxidant potency, especially when compared with other crustaceans, marine organisms, and food sources.

### 2.5. Anti-Inflammatory and Anti-Platelet Properties of TAC and TLC Extracts from All Shrimp Species Assessed

**Figure 1:**
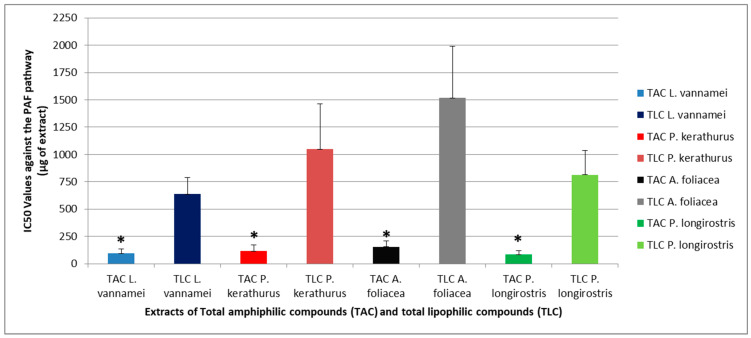
The anti-inflammatory potency of TAC and TLC extracts from all shrimp species, assessed against the PAF-induced thrombo-inflammatory pathway. Biological activity against human platelet aggregation induced via the inflammatory and thrombotic mediator PAF was assessed. Results are expressed as means of the IC_50_ values (half-maximal inhibitory concentrations) in μg of TAC and TLC extract in the aggregometer cuvette that caused 50% inhibition of PAF-induced platelet aggregation in 250 μL of human PRP (the lower the IC_50_ value for a lipid extract, the higher its inhibitory anti-inflammatory effect against PAF). * denotes a statistically significant difference, *p* < 0.05, in the anti-PAF activity of TAC extract from a shrimp species compared to its TLC extract. TAC, total amphiphilic compound; TLC, total lipophilic compound; PAF, platelet activating factor.

**Figure 2:**
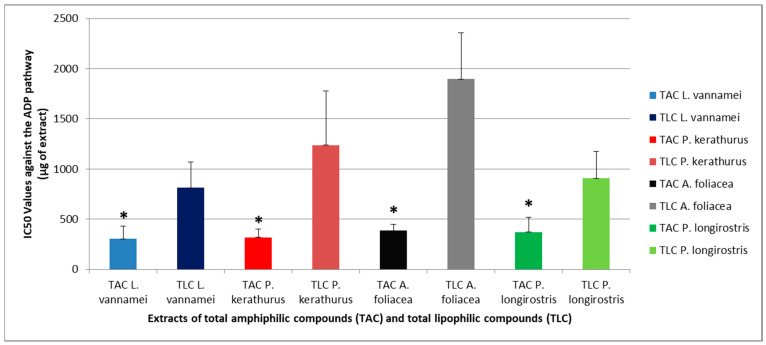
The antithrombotic (antiplatelet) potency of TAC and TLC extracts from all shrimp species assessed against platelet aggregation induced by the thrombotic ADP pathway. Biological activity against human platelet aggregation induced via the classic thrombotic platelet agonist, ADP, was assessed. Results are expressed as means of the IC_50_ values (half-maximal inhibitory concentrations) in μg of TAC and TLC extract in the aggregometer cuvette that caused 50% inhibition of ADP-induced platelet aggregation in 250 μL of human PRP (the lower the IC_50_ value for a lipid extract, the higher its inhibitory antithrombotic (antiplatelet) effect against ADP). * denotes a statistically significant difference, *p* < 0.05, in anti-ADP activity of TAC extract from a shrimp species compared with its TLC extract. TAC, total amphiphilic compound; TLC, total lipophilic compound; ADP, adenosine 5′ diphosphate.

Figure 1 and Figure 2 show an overview of the biological activities (anti-inflammatory and anti-platelet potency, expressed as half maximum inhibitory concentrations; IC_50_ value) of the TAC and TLC extracts of all shrimp species assessed against the thrombo-inflammatory pathway of PAF-induced platelet aggregation of human platelet-rich plasma (PRP), and against platelet aggregation of human PRP via a classic thrombotic platelet agonist (adenosine 5’ diphosphate; ADP), respectively. The results are expressed as means of the IC_50_ values in µg of TACs or TLCs in the aggregometer cuvette causing 50% inhibition of PAF/ADP-induced platelet aggregation. It should be mentioned that a lower IC_50_ value for an extract indicates its higher anti-inflammatory inhibitory effect against the PAF-associated pathway and its antiplatelet inhibitory effect against the specific platelet agonist ADP for platelet aggregation. Additionally, shrimps are rich in bioactive phosphatidylcholines; according to a study conducted by Tsoupras et al., in which a standard marine-derived phosphatidylcholine (PC) bearing DHA within its structure was used as a known compound in bioassays against PAF, thrombin, and ADP pathways under exactly the same conditions that were applied in this study; it was found that such standard PC bioactive molecules rich in n-3 PUFAs showed IC_50_ values (expressed in mean values of μg of standard in the aggregometer cuvette) of 0.3 ± 0.1, 0.6 ± 0.15, and 1.5 ± 0.8 μg against the PAF, thrombin, and ADP pathways, respectively [28]. 

As shown in Figure 1, all the tested extracts exhibited considerable inhibitory activity of similar magnitude against human platelet aggregation induced by the inflammatory and thrombotic PAF pathway of similar magnitude. As far as we know, there has been no previous study evaluating the anti-PAF activity of shrimp extracts on the PAF pathway (as per our research in the Scopus database using the search terms/keywords: shrimp OR prawn AND PAF or “platelet-activating factor”). Within the present study, an evaluation of the anti-inflammatory anti-PAF activities of shrimp extracts is presented for the first time. 

It should also be stressed that PAF constitutes one of the best-known and well established mediators of both inflammation and thrombosis, inducing human cell activation, including platelet activation and aggregation, via binding to its specific G-coupled protein receptor (GCPR) in cell membranes, namely, the PAF receptor (PAFR) [10]. Moreover, the PAF/PAFR thrombo-inflammatory pathway is implicated in several inflammation-related manifestations and associated disorders, including cardiovascular diseases (CVDs) and cancer [10].

Thus, inhibition of this pathway by a compound/extract in a cell model, including human PRP, signifies the strong anti-inflammatory potency of that compound/extract. The platelet aggregation model used in the present study in human PRP is an ideal tool to reveal the substances and their quantities that are capable of attenuating the action of this thrombo-inflammatory factor and, consequently, reducing the risk of diseases related to the activation of platelets and other cells via PAF-related signaling pathways, such as cardiovascular diseases (CVDs) [10,29]. Therefore, the ability of shrimp extracts rich in marine bioactives to inhibit the PAF pathway in human platelets, as observed in the present study, further supports their anti-inflammatory potential.

Interestingly, as shown in Figure 1, the TAC extracts of all shrimp species assessed showed much more potent anti-inflammatory potency against the PAF pathway in human in comparison to the relevant activity of their TLC extracts (*p* < 0.05). It is important to state here that as aforementioned, the separation of the more amphiphilic lipid compounds from shrimps, such as their PLs, based on the extraction process and the counter-current distribution procedure applied in the present study, facilitated their presence within the TAC extracts and their separation from the more lipophilic shrimp lipids like triglycerides, sterols, etc., which migrated to their TLC extracts. PLs from several marine sources have been found to strongly inhibit this thrombo-inflammatory pathway, with several subsequent anti-inflammatory health benefits [1,30], further supporting the anti-inflammatory anti-PAF potential of shrimp TAC extracts rich in marine PLS [10] for future nutraceutical and drug-related applications against thrombosis, inflammation, and associated disorders. 

Moreover, all the tested extracts also exhibited considerable inhibitory activity against human platelet aggregation induced by the classic platelet agonist, ADP, which is also a potent thrombotic agent that acts mostly through its specific P2Y12 and P2Y1 GCPRs in platelets, activation of which also induce cyclooxygenases (COX) and the arachidonic acid pathway of eicosanoid production; thus, it is a drug target against thrombosis [10]. Again, the shrimp TAC extracts rich in marine PLs were more effective against the ADP pathway in comparison to the effect of the shrimp TLC extracts, in all shrimp species assessed (*p* < 0.05), which further emphasizes the potential application of TAC extracts to combat ADP- and COX-related thrombo-inflammatory processes and prevent related diseases. 

Interestingly, the shrimp TAC extracts rich in bioactive marine PLs showed higher specificity against the PAF pathway, since the anti-PAF activity was significantly stronger (lower IC50 values) than their activity against the ADP pathway (higher IC50 values) in all shrimp species assessed (*p* < 0.05 in each comparison of anti-PAF activity versus anti-ADP activity of the TAC within the same shrimp species). Moreover, regarding the anti-PAF activity of the TAC extracts between species, all shrimp TAC extracts showed comparably strong anti-PAF effects (*p* > 0.05). Similarly, when comparing the anti-ADP activity of the TAC extracts between species, all shrimp TAC extracts showed strong anti-ADP effects (*p* > 0.05); however, these were less active in comparison to their anti-PAF effects, as aforementioned. 

These results concerning the stronger anti-PAF effects of marine PLs in comparison to their anti-ADP effects, as observed in the shrimp TAC extracts rich in PLs, and thus, the stronger specificity of those TAC extracts against the PAF pathway in human PRP, are in accordance with previously reported outcomes in other extracts from several other marine species rich in PLs. These include fish like salmon, herring, and other species [1], which usually consume shrimps and microalgae, as well as microalgae themselves [30], which are consumed by shrimps and/or fish. Taking into account that the TAC extracts from all shrimp species showed similar potent anti-PAF activity, as well as less potent but considerable anti-ADP effects of the same magnitude comparable with other healthy marine food sources like salmon, herring, other fish species, and microalgae, the current results further suggest that independently of their origin, all these shrimp species are excellent sources for marine PLs with both strong anti-inflammatory and antithrombotic potency. 

Specifically, n-3 PUFAs, which are esterified to PLs, are released via specific cytoplasmic phospholipase A2 (PLA2). This release in cell membranes promotes the production of anti-inflammatory eicosanoids that act antagonistically to other inflammatory and thrombotic eicosanoids (prostaglandins, leukotrienes, and thromboxanes) and are produced by n-6 PUFAs like arachidonic acid. Additionally, certain dietary PLs with a similar structure to PAF or chemical affinity to its active site can inhibit PAF activity by binding to the PAF receptor (PAFR), which blocks inflammatory signaling. This binding alters the composition and fluidity of specialized microdomains, commonly called lipid rafts, affecting PAFR localization, function, and inflammatory response. Dietary PLs also influence the activation of G protein-coupled receptors (GPCRs), related to inflammatory responses, by altering the balance of eicosanoid production. For example, leukotriene B4 (LTB4) binds to BLT1 and BLT2 receptors, which promotes inflammation. On the other hand, lipoxin A4 (LXA4) binds to the ALX/FPR2 receptor, which promotes anti-inflammatory effects. Thus, by reducing the production of pro-inflammatory eicosanoids through dietary PLs, inflammation can also be reduced [10,15,31].

Since all these samples were assessed in human PRP from healthy volunteers, the current results suggests that these bioactivities seem to provide preventative protection against inflammatory and thrombotic manifestations and associated disorders. Nevertheless, more research is needed in relation to disease state before and after administration of such shrimp-derived TAC extracts rich in marine PL bioactives with anti-inflammatory and antithrombotic potency.

### 2.6. ATR-FTIR Analysis of the TAC Extracts from All Shrimp Species Assessed

ATR-FTIR analysis was performed on the TAC extracts from all the tested shrimp species, since these were the most bioactive samples. The spectra obtained from the ATR-FTIR analysis of all the shrimp-derived TAC extracts showed peaks characteristic of functional groups of phenolics, carotenoids, and marine PLs in all shrimp species, as shown in Table 5. 

In all these shrimp species, the main peaks in the ATR-FTIR spectra that were identified were at 3380 cm^−1^, 2923 cm^−1^, 1735 cm^−1^, 1725 cm^−1^, and 1650 cm^−1^. According to obtained peaks from the spectra of five standard compounds (β-carotene, soy polar lipids, quercetin, catechin, gallic acid), the observed compounds in all the amphiphilic shrimp extracts were β-carotene, polar lipids, and phenolics. Specifically, the presence of β-carotene in the samples was determined from peaks originating from double bonds between carbon atoms (C=C) and sp3 hybridized bonds between hydrogen and carbon, which are commonly found in most types of carotenoids, including β-carotene. Additionally, the presence of polar lipids was also identified for the peaks mentioned previously, found mainly in the hydrophobic fatty acid tails of the amphiphilic molecules. Lastly, the existence of phenolic compounds was determined through the existence of C=C aromatic group peaks and carbonyl group peaks (C=O), although it is important to mention that this evidence does not support the presence of quercetin, catechin, or gallic acid in the shrimp samples, because marine phenolics have complex structures with certain similarities to the functional groups in standard phenolic compounds.

### 2.7. Fatty Acid Composition of the TAC Extracts from All Shrimp Species Assessed

The fatty acid composition of the PLs in the TAC extracts of the samples was determined by liquid chromatography–mass spectrometry (LC-MS) after saponification of all the TAC extracts, and the results obtained are presented in Table 6. 

Remarkably, all PLs in these TAC extracts were found to be very rich in UFAs, which were approximately four times higher in content than the much lower levels of SFAs in all shrimp species (*p* < 0.05). Among these UFAs, the most abundant class was represented by PUFAs, which were more than twice as high in concentration than both the MUFAs and the SFAs in all the shrimp species (*p* < 0.05). Independently of the shrimp species and their origin, among the PUFAs, the most abundant were the n3 PUFAs DHA and EPA, followed by considerable amounts of other n3 PUFAs like docosapentaenoic acid (DPA) and lower but detectable amounts of alpha linolenic acid (ALA), while the most abundant n6 PUFAs were linoleic acid (LA) and arachidonic acid (ARA). In all the studied shrimp species, the main MUFA was oleic acid (OA), while the main SFAs were stearic and palmitic acids. 

These results are in accordance with outcomes following similar trends in previous studies on FAs in PLs and TLs in extracts from several shrimp species, including those studied in the present work [2,6]. Nevertheless, it should also be stressed that the results of the present study further reveal that the TAC extracted from these shrimp species and separated from the other compounds using the specific applied methodology successfully managed to retain the high amounts of unsaturated fatty acids naturally present in the marine PLs in these shrimps, independently of their origin and species. Thus, within the present study, higher overall amounts of UFAs and PUFAs were detected in these shrimp species than in the same or other shrimp species, as measured in their PL or TL extracts [2,6]. 

Moreover, the high n3 PUFA content in the PL bioactives of the TAC extracts was also reflected by their very low n6–n3 PUFA ratio, with values lower than 1.0 in all TAC samples from the shrimp species assessed. Taking into account that the lower the n-6–n-3 PUFA ratio for a source/extract, the greater its prophylactic anti-inflammatory benefits against various chronic disorders related to inflammation and platelet aggregation, and vice versa [38], the very low values for this ratio observed in the PL bioactives present in the TAC extracts from all shrimp species further support their anti-inflammatory and antithrombotic health-promoting potential against inflammation-related manifestations and disorders. Of all the TAC extracts, the PLs of *P. longirostris* had the highest concentration of n3 PUFAs, as reflected by high levels of the main n3 PUFAs, DHA, EPA, and DPA, and the lowest n6 PUFA levels and thus, the lowest n6–n3 PUFA ratio in comparison to the PLs in the TAC extracts from all the other shrimp species (*p* < 0.05).

In addition, the high n3 PUFA content detected in the shrimp-derived PLs also provides an explanation for the potent anti-inflammatory and antithrombotic bioactivity observed for these TAC extracts from all the shrimp species, assessed against both the PAF and ADP pathways. PLs from other marine sources with high concentrations of UFAs and especially, n3 PUFAs have also shown strong anti-inflammatory and antithrombotic bio-functionalities against the PAF, thrombin, and ADP pathways of platelet activation and thrombo-inflammation [1]. 

Similar outcomes were also observed in the unsaponified fractions of all the TAC extracts from these shrimp species, which contained the free fatty acids that were released during mechanical breakdown and/or lipase/phospholipase enzymatic activities that may have taken place during the blending process (prior to the extraction) and migrated into the TAC extracts during the extraction and separation processes. Nevertheless, FFAs reflected a very low portion of the fatty acid composition of the TAC extracts, as the majority of the FAs in these extracts were bound to PL and needed saponification for their release and quantification. Within the present study, we also quantified low amounts of the FFA present in the TAC extracts; the analysis is shown in Table 7.

In summary, all FFAs present in the TAC extracts were again found to be very rich in UFAs, especially n3 PUFAs like DHA and EPA, followed by the n6 PUFAs LA and ARA, with very low levels for the n6–n3 PUFA ratio (again, the values for this ratio were close to 1.0 in all TAC of the shrimp species assessed), further supporting the anti-inflammatory potential of the TAC extracts, even though the FFAs contributed little to the overall FA composition of these shrimp-derived TAC extracts (the majority of the FAs were bound to PLs within these TAC extracts). 

### 2.8. LC-MS Analysis and Structural Elucidation of the Marine PL Bioactives Present in the TAC Extracts of All Shrimp Species Assessed

This analysis focused on the structure of phospholipids, specifically, phopsphatidylcholines (PC) and phosphatidylethylamines (PE), as several other LC-MS/MS studies on marine organisms, including shrimps, also reported that the vast majority of the polar lipids in such extracts consisted mainly of PC and PE [39,40]. Additionally, it has been proven by many studies that several types of PC and PE, either alkyl–acyl or diacyl, with n3PUFAs at the *sn*-2 position in their glycerol backbone of their structures, exert significant activity against inflammation and thrombosis-related disorders by acting as antagonists, agonists, or inhibitors in several thrombo-inflammatory pathways, especially the PAF/PAF-receptor pathway [1]. 

As shown in Table 8, in the present study, in the PL of the TAC extracts of *L. vannamei*, diacyl PCs, alkyl–acyl PCs, diacyl PEs, and alkyl–acyl PEs were observed to exist, mainly containing PUFAs within their structures, whereas the most abundant unsaturated fatty acid, at the *sn*-2 position of their glycerol backbone, was mainly identified as EPA (20:5 n-3), followed by DHA (C22:6 n-3), ARA (C20:4 n-6), LA (C18:2 n-6), and other UFA (C18:3, 20:2, 22:5), in accordance with the literature. An additional noteworthy FA that belongs to the MUFA category is palmitoleic acid (C16:1), located at the sn-1 position in most of the recognized compounds and mostly present in the PE subclasses. Amongst the identified polar lipids, only a few of them contained SFAs in their structures, as validated by the fatty acid composition, whereas SFAs were present in a significantly lower percentage compared with the other classes (MUFAs, PUFAs). Nevertheless, palmitic acid (PA, C16:0) was the only fatty acid found exclusively in the PE subclass, mostly at the *sn*-1 position.

Furthermore, *P. Kerathurus* TAC extracts contained PL that showed similar results to the first shrimp species, with PUFAs being the most prevalent class of fatty acids in both polar lipid subclasses. In particular, DHA (C22:6 n-3) and DPA (22:5 n-3) were identified equally in diacyl and alkyl–acyl PC and PE molecules as abundant fatty acids. 

Other noteworthy FAs were EPA (C20:5 n3), ARA (C20:4 n6), and ardenic acid (C22:4 n-6), while LA (C18:2) and α-linoleic acid (C18:3) were noticed less. Several types of monounsaturated fatty acids were observed from the structural elucidation of the polar lipids. Specifically, OA (C18:1), palmitoleic (C16:1), and gadoleic (C20:1) acid were found in these PLs. Additionally, the SFAs recorded in the PLs of this shrimp species were palmitic (C16:0) and stearic acid (C18:0) in both the PE and PC bioactives.

In *A. foliacea*, the prevalent fatty acids in the PLs of its TAC extracts were again PUFAs, with the most representative being the DHA (C22:6 n-3), EPA (C20:5 n-3), DPA (C22:5 n-3), ARA (C20:4 n-6), LA (C18:2 n-6), and dihomolinoleic (C20:2 n-6) and dihomolinolenic acid (C20:3 n-6). Moreover, in the PE and PC from these TAC extracts, the main MUFAs attached to the glycerol backbone were gadoleic (C20:1), palmitoleic (C16:1), and OA (C18:1 n-9). A couple of the identified polar lipids also contained SFAs, specifically PA (16:0) and OA (18:0), at the sn-1 position of their glycerol backbone in both substances.

Once again, the TAC extracts of *P. longirostris* consisted majorly of PE and PC with PUFAs attached to their glycerol backbones. According to the analysis, DHA (C22:6 n-3) was the most abundant fatty acid in these PL bioactives, followed by EPA (C20:5 n-3), DPA (C22:5 n-3), ARA (C20:4 n-6), ardenic (C22:4 n-6), and linoleic acid (C18:2 n-6). Furthermore, in all PL subclasses, the most frequent MUFAs were oleic (C18:1 n-9), palmitoleic (C16:1), and gadoleic acid (C20:1), respectively. Similar to the previous shrimp species, SFAs were found to be more consistent; palmitic acid (C16:0) was the most common fatty acid in this category, while stearic acid (C18:0) was also observed.

Briefly, in all the shrimp species examined, it was found that PUFAs were the most common fatty acid class linked to the glycerol backbone of the main polar lipid bioactives, PC and PE. This observation is consistent with other studies that have examined the structural elucidation of the polar lipids from several shrimp species [39,41,42,43] and other marine organisms. Thus, the most abundant fatty acids found in the bioactive PCs and PEs in TAC extracts from shrimps are the n-3 PUFAs DHA (C22:6 n-3) and EPA (C20:5 n-3), providing an explanation for the structural activity of these PL bioactives against thrombo-inflammatory signaling related to PAF, ADP, and eicosanoids. The presence of MUFAs was also frequently noted, with the main fatty acids in this category being palmitoleic (C16:1), oleic (C18:1 n-9), and gadoleic acid (C20:1). Taking into account that consistent SFAs were observed only in the final shrimp species examined, this category was the least likely to be found attached to the structures of PE and PC biomolecules, with palmitic (C16:0) and stearic (C18:0) being the fatty acids that were identified.

## 3. Materials and Methods

### 3.1. Samples, Materials, Reagents, and Instrumentation

Samples of wild mature individuals of four different shrimp species, *Litopenaeus vannamei*, *Penaeus kerathurus*, *Aristaeomorpha foliacea*, and *Parapenaeus longirostris*, were retrieved from the Fisheries Research Institute of Nea Peramos, Kavala, Greece. 

All reagents (Folin–Ciocalteu, DPPH, ABTS), solvents (chloroform, methanol, petroleum ether, ethanol, n-octane, isopropanol), phenolics (Trolox, gallic acid, quercetin, catechin), and lipid standards (soybean polar lipids, β-carotene) were purchased from Sigma Aldrich (St Louis, MO, USA). UV–Vis spectroscopy analyses were performed on an LLG-uniSPEC 2 spectrophotometer (Am Hambuch 1 53340 Meckenheim Germany)and ATR-FTIR spectroscopy on a Perkin Elmer Frontier ATR/FT-NIR/MIR spectrometer (Perkin Elmer, Shelton, CT, USA).

All plastic consumables, reagents, and solvents used in the antiplatelet assays were of analytical grade, purchased from Sigma Aldrich (St Louis, MO, USA). The 20-gauge (G) safety needles and evacuated sodium citrate S-monovettes^®^ for blood sampling were purchased from Sarstedt Ltd. (Wexford, Ireland). Bioassays on human PRP (hPRP) were performed on a Chrono-log 490 four-channel strobilometric platelet aggregometer (Havertown, PA, USA), connected to the accompanying AGGRO/LINK®8 program software package. All consumables for platelet aggregation were purchased from Chrono-log (Havertown, PA, USA). PAF, ADP, and bovine serum albumin (BSA) standards were purchased from Sigma Aldrich (St Louis, MO, USA). Centrifugations were performed on a 4000 rpm maximum-capacity Nahita Blue Medibas+ low-speed centrifuge from Auxilab (Beriain, Navarra, Spain).

### 3.2. Extraction of Lipid Bioactives from All Shrimp Species and Separation into Their Total Amphiphilic (TAC) and Total Lipophilic Content (TLC)

Total lipids (TLs) of approximately 100g of shrimp from each species were obtained by extraction based on a modification of the method described by Bligh and Dyer [13] according to Tsoupras et al. [15]. Each of the obtained TL samples was further separated into its total lipophilic content (TLC) and total amphiphilic content (TAC) by counter-current distribution, based on the methodology presented by Galanos and Kapoulas [14] as described by Tsoupras et al. [15,29]. 

Briefly, extraction of TLs was performed by blending and thereby homogenizing the whole of each shrimp sample (head, shell and flesh) in a single-phase system containing chloroform–methanol–water at a ratio of 1:2:0.8 (*v*/*v*/*v*). The homogeneous solution was then filtrated in a Buchner apparatus with a filter paper, and the filtrate was then transferred to a separation funnel, to which water and chloroform were added in appropriate volumes to adjust the homogenate to a chloroform–methanol–water ratio of 1:1:0.9 (*v*/*v*/*v*) to achieve phase separation. The lower phase (chloroform) containing the TL extracts was then collected in spherical flasks and the solvents (mainly chloroform) were evaporated in a rotary evaporator under vacuum. The TLs were obtained from the spherical flasks by re-dissolving them in small volumes (approx. 1–5 mL) of 1/1 (*v*/*v*) chloroform/methanol solution, facilitating their transfer into small preweighed glass tubes, which were further evaporated under nitrogen stream.

Each of the TL samples was then further separated into TLC and TAC by counter-current distribution based on pre-equilibrated petroleum ether (for obtaining TLC extracts) and 87% ethanol in water (for obtaining TAC extracts). Solvents from the separated TLC and TAC extracts were evaporated again in a rotary evaporator Rotavapor R-300 (Flawil, Switzerland) under vacuum and then transferred to small preweighed glass tubes, using small volumes (1–3 mL) of petroleum ether for TLC extracts and 1/1 (*v*/*v*) chloroform/methanol solution for TAC extracts.

The solvents used to transfer the extracted lipids into the tubes were evaporated under a stream of nitrogen. The obtained TL, TAC, and TLC extracts from each sample were weighed to obtain the extraction yield and then stored in the freezer for further analysis. 

The percentages of the extraction yields were calculated via the following equation:

**Extraction yield (%) = (Mass of dried extract (mg)/Mass of shrimp sample (mg)) × 100%**

For all the subsequent analyses, each of the dry TAC extracts was dissolved in 1 mL of ethanol, while each dry TLC extract was dissolved in 1 mL of octane. Then, all were equally aliquoted into individual glass tubes, and all the solvents were again evaporated under nitrogen stream to obtain dry TAC and TLC samples for further analysis.

### 3.3. Total Carotenoid Content (TCC) Analysis

The determination of the total carotenoid content (TCC) in each extract was performed according to the method described by Tsoupras et al. [15]. Briefly, each aliquoted sample was dissolved in 2 mL of octane and its absorbance at 450nm was then measured. The concentration sought was measured based on the β-carotene standard curve and the obtained results were expressed in mg β-carotene equivalent (CE)/g extract.

### 3.4. Total Phenolic Content (TPC) Analysis

The total phenolic contents (TPCs) of all the extracts were evaluated using Folin–Ciocalteu reagent. Specifically, 1 mL of distilled water and 1 mL of Folin–Ciocalteu reagent were added to each sample according to the method described by Tsoupras et al. [15]. After 7 min, 3 mL of Na_2_CO_3_ was transferred to each sample. This was followed by incubation of the samples in a dark place for 2 h. Between the successive additions of reagents and every 30 min during the incubation, the solutions were vortex stirred. After the two-hour incubation of the samples, their absorbance at 765 nm was measured. The concentration sought was measured based on a gallic acid-based standard curve and the results obtained were expressed in mg gallic acid equivalent (GAE)/g extract.

### 3.5. Assessment of Antioxidant Activities of Extracts

The evaluation of the antioxidant activity of the samples was carried out via three different assays: the 1,1-diphenyl-2-picrylhydrazyl (DPPH) radical scavenging assay; the ABTS (2,2′-azinobis-(3-ethylbenzothiazoline-6-sulfonic acid)) radical cation decolorization method; and the ferric-reducing antioxidant power (FRAP) method, according to Tsoupras et al. [15] and Xiao et al. [21].

Briefly, for the DPPH assay, 0.2 mL of ethanol, 0.8 mL of Tris-HCl buffer (pH 7.4), and 1 mL of DPPH solution were transferred to each sample. Between successive additions of the reagents, the solutions were vortex-stirred. The solutions were left at room temperature for 30 min and immediately afterwards, their absorbance at 517nm was recorded. Trolox was used as positive control, and a mixed solution with 1.2 mL of ethanol and 0.8 mL of Tris-HCl buffer was used as the blank. The percentage of inhibition (%) was calculated from the following equation:

**Inhibition (%) = (A1 − A2) × 100/A1,**

where A1 is the absorbance of the control sample solution and A2 is the absorbance of the test sample solution.

The IC_50_ value, i.e., the concentration of each extract with the ability to neutralize 50% of the DPPH radical, was then calculated. The DPPH radical scavenging activity of the sample was expressed as Trolox-equivalent antioxidant capacity (TEAC). TEAC was calculated as follows:

TEAC = IC_50_ of Trolox (μg/L)/IC_50_ of the sample (μg/L).

For the ABTS assay, 2 mL of ABTS solution was transferred to each sample, followed by vortex stirring. The solutions were incubated in darkness for 7 min and immediately afterwards, their absorbance at 743nm was measured. Trolox was used as a positive control, while distilled water was the blank. The concentration of Trolox was chosen under the conditions of an absorbance value ranging from 0.2 to 0.8 for the standard curve. 

The results were expressed as μmol TE/g DW, according to the following formula:

**ABTS (μmol TE/g DW) = c × V × t/m,**

where c is the Trolox concentration (µmol/mL) of the corresponding standard curve of the diluted sample, V is the volume of the sample (mL), t is the dilution factor, and m is the dry weight of the sample (g).

For the FRAP assay, 3 mL of FRAP solution was transferred to each sample, followed by vortex stirring. The solutions were incubated in darkness at 37 °C for 15 min and immediately afterwards, their absorbance at 593nm was measured. Trolox was used as positive control and distilled water as blank. The concentration of Trolox was chosen under the conditions of an absorbance value ranging from 0.2 to 0.8 for the standard curve.

The results were expressed as μmol TE/g DW, according to the following formula:

**FRAP (μmol TE/g DW) = c × V × t/m,**

where c is the Trolox concentration (µmol/mL) of the corresponding standard curve of the diluted sample, V is the volume of the sample (mL), t is the dilution factor, and m is the dry weight of the sample (g).

### 3.6. Assessment of Anti-Platelet and Anti-Inflammatory Properties of Extracts via Light-Transmitance Aggregometry

The bioassays for evaluating the anti-platelet and anti-inflammatory properties of all extracts against the PAF-pathway and their antithrombotic effects against the ADFP pathway were performed in human platelet-rich plasma (hPRP) preparations from healthy donors, assessing their capacity to inhibit the aggregation of human platelets when hPRP was induced either via the inflammatory and thrombotic mediator PAF or via the well-established platelet agonist ADP, in the presence of these extracts, as previously described by Tsoupras et al. [15]. According to the method developed by Tsoupras and others, a total of 50 mL of blood was collected using a 20 G safety needle. The collected blood was added via sodium citrate anticoagulant S-monovettes and then, the blood samples were centrifuged at 194× *g* for 18 min with no break applied, in order to prevent platelet aggregation. After the centrifugation, the supernatant hPRP was transferred to polypropylene tubes using Pasteur pipettes. The platelet-poor plasma (PPP) was centrifuged at 1465× *g* for 20 min at 24 °C with no break applied. hPRP was adjusted to 500,000 platelets/μL if required by adding the respective volume of PPP according to the absorbance of the hPRP measured in the spectrophotometer. All analyses were completed within 2.5 h of the initial blood draw, and all procedures were conducted at 24 °C. The platelet-activating factor (PAF) used in the analysis was a standard solution dissolved in chloroform/methanol (1:1 *v*/*v*); the aliquoted solutions were evaporated under a nitrogen stream and re-dissolved in bovine serum albumin solution (BSA). Shrimp TAC and TLC samples were also dissolved in BSA (2.5 mg BSA/mL saline). Standard active ADP was dissolved in saline prior to testing. By adding different concentrations of each sample to the platelet suspension, the capacity of each sample to inhibit either ADP-induced or PAF-induced platelet aggregation was investigated. Before testing, 250 µL of hPRP was added to an aggregometer cuvette at 37 °C while being stirred at 1200 rpm and then calibrated with a blank PPP. A linear curve was deduced for the 20–80% inhibition range against PAF-induced/ADP-induced aggregation of hPRP for each sample concentration. From this curve, the 50% inhibitory concentration value, or IC_50_ value, for each sample was determined as the concentration of the sample that resulted in 50% of PAF-induced/ADP-induced aggregation of hPRP [15,29]. The anti-inflammatory and antithrombotic potencies of each sample were expressed as means of their IC_50_ values (half-maximal inhibitory concentrations) ± standard deviation (SD), quantified in mass (μg) of the bioactive TAC or TLC extract present in the aggregometer cuvette that caused 50% inhibition of hPRP aggregation induced by either PAF or ADP. Each sample was assessed several times in blood samples from different healthy donors (N = 6) to ensure reproducibility.

### 3.7. ATR-FTIR Analysis

In order to capture the ATR-FTIR spectra of our samples and of the five standards (β-carotene, gallic acid, catechin, quercetin, and polar lipids), a Perkin Elmer Frontier ATR/FT-NIR/MIR spectrophotometer was used according to Vordos et al. [44]. Dried TAC extracts were placed in the spectrometer and spectra were recorded from 4000 to 600 cm^−1^ using 32 scans. All spectra were corrected against the air background spectrum.

### 3.8. LC–MS Analysis

Liquid chromatography–mass spectrometry (LC-MS) was used to quantify the fatty acid composition of all extracts and to evaluate the overall structures of the PLs in the TAC extracts, as described by Tsoupras et al. [1,45]. Briefly, each extract was re-dissolved in 500 μL dichloromethane/methanol (1:2, *v*/*v*) and then centrifuged for 6 min at 13,000 rpm (Heraeus Biofuge Stratos, Fisher Scientific Ltd., Dublin, Ireland). The supernatant was then filtered through Sartorius Minisart Syringe Filters (0.2 µm, 15 mm PTFE). In order to obtain the fatty acid profiles of these filtrates, 10 μL of each filtrate was injected into an HPLC system (Agilent 1260 series, Agilent Technologies Ireland Ltd., Little Island, Co., Cork, Ireland) equipped with a Q-TOF mass spectrometer (Agilent 6520, Agilent Technologies Ireland Ltd., Little Island, Co., Cork, Ireland) using electrospray ionization (ESI) as the source type. Separation of fatty acids was achieved using an Agilent C18 Poroshell 120 column (2.7 μm, 3.0 × 150 mm) (Agilent Technologies Ireland Ltd., Little Island, Co., Cork, Ireland) with gradient elution, in which mobile phase A consisted of 2 mM ammonium acetate in water and mobile phase B consisted of 2 mM ammonium acetate in 95% acetonitrile. The mobile phase had an initial flow rate of 0.3 mL/min until 5 min elapsed; this was increased to 0.6 mL/min after 10 min. The flow was maintained at this rate until the end of the run. The mass spectrometer scanned m/z values from 50 to 1100, while reference masses 1033.988 and 112.9855 were used to monitor the scan in negative ionization mode. The capillary voltage was 3500 V and the skimmer and fragmenter voltages were maintained at 65 V and 175 V, respectively. The drying gas flow, pressure, and temperature of the nebulizer were set to 5 L/min, 30 psi, and 325 °C, respectively.

Validation of the LC-MS method was performed by comparing the specific accurate masses and retention time (RT) of various standards, such as saturated and unsaturated fatty acids, namely lauric (C12:0), myristic (C14:0), palmitic (C16:0), stearic (C18: 0), oleic (OA, C18:1n-9 cis), linoleic (LA, C18:2 n-6 cis), gamma-linolenic (GLA, C18:3 n-6), α-linolenic (ALA, C18:3 n-3), arachidonic (ARA, C20: 4 n-6), eicosapentaenoic (EPA, 20:5 n-3), docosahexaenoic (DPA, 22:5 n-3), and docosahexaenoic acid (DHA, 22:6 n-3) (Sigma, Wicklow, Ireland). The peak area of each identified fatty acid was the average of triplicate sample injections, and their relative content was recorded according to their average peak area. Since the peak areas do not reflect the exact amounts of individual fatty acids, the relevant data should be read with caution.

The assignment of FFA and phospholipid species was based upon a combination of survey, daughter, precursor, and neutral loss scans, and the identity of the bioactive PL molecules was verified using the LIPID MAPS: Nature Lipidomics Gateway (www.lipidmaps.org, accessed on 21 October 2024), with reference to the lowest delta values combined with the results obtained from the LC-MS analysis of the fatty acid composition of the saponified PLs, as previously described by Tsoupras et al. [1,45].

### 3.9. Statistical Analysis

The Kolmogorov–Smirnov criterion was assessed for all data for each assay/determination/quantification, to test normality (when *p* > 0.05, values were normally distributed; when *p* < 0.05, values were not normally distributed). One-way analysis of variance (ANOVA) was used for all comparisons of IC50 values against PAF-induced aggregation of human platelets, as they followed a normal distribution, while Kruskal–Wallis nonparametric multiple comparison tests were used for all the other comparisons that did not follow a normal distribution. Differences were considered to be statistically significant when the *p* value was less than 0.05 (*p* < 0.05). The data were analyzed using a statistical software package (IBM-SPSS Statistics 26 for Windows, SPSS Inc., Chicago, IL, USA).

## 4. Conclusions

This is the first study demonstrating that TAC extracts from shrimps contain bioactive amphiphilic compounds with potent anti-inflammatory effects against the thrombo-inflammatory mediator PAF and also exert antiplatelet activity against the well-established platelet agonist ADP in human blood samples, independently of shrimp species. Structural analysis and quantification of samples revealed that these shrimp-derived TAC extracts were rich in PL bioactives, such as PCs and PEs containing long-chain n3 PUFAs like DHA and EPA within their structures, providing a structurally active relationship for their strong anti-inflammatory and antithrombotic health-promoting properties against the PAF pathway and the ADP/eicosanoid pathways, also further supported by the favorable, very low values in the n6/n3 PUFA ratio observed in these PLs. Moreover, all the shrimp lipid extracts were also rich in marine phenolics and carotenoids, a result associated with the observed strong antioxidant capacities of the shrimp-derived TAC extracts. Conclusively, all shrimp species possessed promising and comparable antioxidant, anti-inflammatory, and antithrombotic properties; however, the TAC extracts from the Thracian *P. longirostris* apparently provided the most beneficial outcomes with respect to anti-inflammatory and antithrombotic protection, as their PL bioactives contained statistically significant higher n-3 PUFA, EPA, and DHA content and thus, lower n6/n3 ratios, further supporting the stronger anti-inflammatory and antithrombotic activities of the TAC extracts from this shrimp species.

Overall, shrimp seem to be an excellent source of bioactive TAC extracts that are rich in marine bioactive PLs with anti-inflammatory and antithrombotic properties, as well as in antioxidant phenolics and carotenoids, all of which can further be valorized as bio-functional ingredients in several nutraceutical and pharmaceutical applications and products with health-promoting properties against thrombo-inflammatory manifestations and associated disorders.

## 5. Future Perspectives

As there has been insufficient research on shrimp lipids, the present study unveiled the health benefits that shrimp lipid extracts have on inflammation, thrombosis, and oxidative stress, and especially, amphiphilic bioactives from shrimp rather than their lipophilic contents. Nevertheless, more research is needed in such amphiphilic shrimp extracts derived with green extraction techniques to obtain amphiphilic lipid bioactives separately from shrimp meat and shrimp by-products (i.e., heads). This research should be carried out in accordance with EU regulations on food-grade extraction solvents, in order to promote environmentally friendly and sustainable techniques that can be applied in industry, while at the same time, following circular economy guidelines as mandated by the UN. This will address the major environmental issue of shrimp waste disposal and reveal the advantages of processing shrimp by-products, leading to sustainable utilization and circular economy within a green technology framework. Furthermore, clinical trials and sensory tests should be conducted with several applications of these shrimp TAC bioactives as bio-functional ingredients in several functional products like functional foods, supplements, nutraceuticals, cosmeceuticals, cosmetics, pharmaceuticals, and drugs, thus assessing their health-promoting properties in an in vivo setting, according to the Declaration of Helsinki. This type of research will provide further insight into both the preventative and the therapeutic potential of the amphiphilic bioactives from shrimp and shrimp by-products.

## Figures and Tables

**Table 1:** Yield of TAC, TLC, and TL extraction from all shrimp species, expressed as g of dried extract/100 g of shrimp.

Yield of Extraction				
		g of Dried Extract/100 g Shrimp		
Shrimp Species	Extract	Median	Minimum	Maximum
*L. vannamei*	TAC	0.19 *	0.11	0.24
	TLC	0.07	0.03	0.07
	TL	0.22	0.18	0.31
*P. kerathurus*	TAC	0.54 *	0.52	0.74
	TLC	0.20	0.13	0.21
	TL	0.75	0.65	0.96
*A. foliacea*	TAC	1.41	0.88	1.48
	TLC	1.02	0.77	1.27
	TL	2.50	1.66	2.68
*P. longirostris*	TAC	0.73 *	0.35	0.76
	TLC	0.11	0.10	0.21
	TL	0.87	0.45	0.94

Abbreviations: TAC, total amphiphilic compound; TLC, total lipophilic compound; TLs, total lipid; * denotes statistically significant differences in TAC extract yield compared with TLC extract from the same shrimp species (*p* < 0.05).

**Table 2:** Total carotenoid contents in TAC, TLC, and TL extracts from all shrimp species (expressed as CE per g of dried extract).

Total Carotenoid Content (TCC)				
		CE per g of Dried Extract		
Shrimp Species	Extract	Median	Minimum	Maximum
*L. vannamei*	TAC	0.902	0.782	1.000
	TLC	9.813	5.821	14.237
	TL	10.871	6.603	15.139
*P. kerathurus*	TAC	0.680	0.562	0.683
	TLC	8.900	5.087	14.483
	TL	9.579	5.650	15.167
*A. foliacea*	TAC	1.473	1.072	2.720
	TLC	7.270	5.538	9.692
	TL	8.743	6.610	12.412
*P. longirostris*	TAC	0.909	0.409	1.082
	TLC	8.864	8.731	9.590
	TL	9.640	9.273	10.672

Abbreviations: CE, β-carotene equivalent; TAC, total amphiphilic compound; TLC, total lipophilic compound; TL, total lipid.

**Table 3:** Total carotenoid contents in TAC, TLC, and TL extracts from all shrimp species (expressed as CE per g of dried extract).

Total Phenolic Content (TPC)				
		GAE per g of Dried Extract		
Shrimp Species	Extract	Median	Minimum	Maximum
*L. vannamei*	TAC	10.403	5.408	11.458
	TLC	ND	-	-
	TL	10.403	5.408	11.458
*P. kerathurus*	TAC	4.401	3.755	6.295
	TLC	ND	-	-
	TL	4.401	3.755	6.295
*A. foliacea*	TAC	2.080 *	1.876	2.904
	TLC	ND	-	-
	TL	2.080	1.876	2.904
*P. longirostris*	TAC	2.569	2.193	7.620
	TLC	ND	-	-
	TL	2.569	2.193	7.620

Abbreviations: GAE, gallic acid equivalent; TAC, total amphiphilic compound; TLC, total lipophilic compound; TL, total lipid; * denotes statistically significant difference between the lowest TPC content in the TAC (and thus TL) extracts of this shrimp species compared with the TPC content of the TAC (and thus TL) extracts of the other shrimp species (*p* < 0.05).

**Table 4:** Anti-oxidant activities of the TAC, TLC, and TL extracts from all shrimp species.

		TEAC (DPPH)			ABTS Value (μmol TE/g DW)			FRAP Value (μmol TE/g DW)		
		Median	Minimum	Maximum	Median	Minimum	Maximum	Median	Minimum	Maximum
*L. vannamei*	TAC	0.003	0.001	0.007	7.2	3.8	7.9	2060.5	1403.4	3123.8
	TLC	0.011 *	0.006	0.021	73.7 *	29.3	152.1	2347.2	1058.9	3259.1
	TL	0.014	0.013	0.021	80.8	37.3	155.9	5319.6 ^#^	2462.3	5471.1
*P. kerathurus*	TAC	0.001	0.001	0.002	2.8	2.2	27.4	ND	-	-
	TLC	0.004 *	0.002	0.008	16.5	9.5	23.8	2773.7	1967.9	5848.6
	TL	0.005	0.004	0.009	26.6	18.7	36. 9	2773.7 ^#^	1967.9	5848.6
*A. foliacea*	TAC	0.0003	0.000005	0.0005	9.5	1.1	19.5	292.3	219.5	365.1
	TLC	0.0005	0.0004	0.0012	2.4	1.4	5.9	ND	-	-
	TL	0.0009	0.0005	0.0015	15.4	2.5	22.0	292.3	219.5	365.1
*P. longirostris*	TAC	0.002	0.001	0.002	8.4	5.4	9.8	ND	-	-
	TLC	0.005	0.003	0.008	30.0	2.0	56.4	1745.8	165.2	1891.5
	TL	0.006	0.005	0.009	38.4	7. 4	66.2	1745.8	165.2	1891.5

Abbreviations: TE, Trolox equivalent; DW, dry weight of extract; GAE, gallic acid equivalent; TAC, total amphiphilic compound; TLC, total lipophilic compound; TL, total lipid; ND, not detected; * indicates the statistically significant difference (*p* < 0.05) of the highest ABTS value detected in the TLC extract from the first shrimp species compared with the relevant lower value of the TLC extract from the second and third shrimp species. ^#^ denotes statistically significant differences (*p* < 0.05) between the higher FRAP values of the two first shrimp species and the relevant lower FRAP values of the TL extracts from the other two shrimp species.

**Table 5:** Functional groups of specific bioactives detected via ATR-FTIR analysis of shrimp-derived TAC extracts.

Functional Group	cm^−1^	Bioactive Compound	Reference
O-H (stretching vibration)	3378–3380	Marine phenolics	[32,33]
C-H sp^3^	2921–2926	Carotenoids/polar lipids	[34,35,36]
C=C	1731–1749	Carotenoids/polar lipids	[34,35,36]
C=O	1701–1754	Phenolics	[33]
C=C aromatic group	1645–1658	Phenolics	[37]

**Table 6:** Fatty acid profiles of the PLs present in TAC extracts (saponified samples) from all the shrimp species, expressed as means for each FA (with the relevant standard deviation; SD); percentage composition of the total fatty acids in each extract assessed (n = 3).

Fatty Acid Emperical Name	Type of Fatty Acid (Carbon Atoms, Double Bonds, and Their Positions)	*L. vannamei*		*P. kerathurus*		*A. foliacea*		*P. longirostris*	
		Mean	SD	Mean	SD	Mean	SD	Mean	SD
Myristic	C14:0	0.3	0.1	0.3	0.1	0.2	0.1	ND	-
Pentadecylic	C15:0	0.2	0.1	0.5	0.1	0.2	0.1	0.2	0.1
Palmitic	C16:0	15.5	0.7	11.0	0.1	14.4	0.4	10.5	0.1
Palmitoleic	C16:1 c9 (n7 MUFA)	1.0	0.1	3.9	0.1	5.5	0.2	4.1	0.1
Margaric	C17:0	1.2	0.1	1.8	0.1	0.9	0.1	0.8	0.1
Stearic	C18:0	11.0	0.2	6.2	0.1	8.0	0.3	3.8	0.1
OA	C18:1 c9 (n9 MUFA)	17.4	1.2	14.9	0.2	12.3	0.6	22.2	0.1
LA	C18:2 c9,12 (n6 PUFA)	17.4	2.1	1.2	0.1	2.8	0.1	0.7	0.1
ALA	C18:3 c9,12,15 (n3 PUFA)	1.3	0.1	0.7	0.1	0.3	0.1	0.2	0.1
Stearidonic	C18:4 c6,9,12,15 (n3 PUFA)	0.1	0.1	0.2	0.1	0.1	0.1	0.2	0.1
Nonadecylic	C19:0	0.1	0.1	0.2	0.1	0.4	0.1	0.1	0.1
Gadoleic	C20:1 c9 (n11 MUFA)	1.3	0.1	3.0	0.1	14.6	0.5	1.5	0.1
Dihomolinoleic	C20:2 c10,12 (n6 PUFA)	3.8	0.1	1.1	0.1	2.2	0.1	0.9	0.1
Dihomolinolenic	C20:3 c8,11,14 (n6 PUFA)	0.4	0.1	0.4	0.1	1.2	0.1	0.3	0.1
ARA	C20:4 c5,8,11,14 (n6 PUFA)	3.5	0.1	14.0	0.2	10.6	0.6	7.4	0.1
EPA	C20:5 c5,8,11,14,17 (n3 PUFA)	11.0	0.6	18.0	1.0	7.6	0.6	20.0 ^#^	0.1
Docosadienoic	C22:2 c13,16 (n6 PUFA)	ND	-	0.3	0.1	0.2	0.1	0.2	0.1
Eranthic	C22:3 c5,13,16 (n6 PUFA)	ND	-	0.1	0.1	0.1	0.1	0.1	0.1
Ardenic	C22:4 c7,10,13,16 (n6 PUFA)	0.1	0.1	1.5	0.1	1.0	0.1	0.7	0.1
DPA	C22:5 c7,10,13,16,19 (n3 PUFA)	1.4	0.1	4.0	0.1	7.0	0.2	2.4	0.1
DHA	C22:6 c4,7,10,13,16,19 (n3 PUFA)	12.9	1.3	16.7	0.2	10.5	0.6	23.7 ^#^	0.1
SFA		28.3	0.9	20.0	0.1	24.0	0.7	15.5	0.1
UFA		71.7 **	0.9	80.0 **	0.1	75.9 **	0.7	84.5 **	0.1
MUFA		19.8	1.2	21.8	0.3	32.3	0.7	27.8	0.1
PUFA		51.9 *	1.8	58.2 *	0.4	43.6 *	1.2	56.7 *	0.1
n3PUFA		26.7	0.6	39.6	0.7	25.5	0.9	46.5 ^#^	0.1
n6PUFA		25.2	2.0	18.6	0.3	18.1	0.3	10.2	0.1
n6/n3		0.9	0.1	0.5	0.1	0.7	0.1	0.2 ^#^	0.1

** denotes statistically significant difference (*p* < 0.05) in UFAs compared with SFAs; * denotes statistically significant difference (*p* < 0.05) in PUFAs compared with MUFAs; ^#^ denotes statistical significant difference between the values of EPA, DHA, n3 PUFAs, and n6/n3 ratio of *P. longirostris* compared with these values for other shrimp species. Abbreviations: n3 = omega-3; PUFA = polyunsaturated fatty acid; n6, omega-6; MUFA = monounsaturated fatty acid; SFA = saturated fatty acid; OA = oleic acid; ALA = alpha linolenic acid; LA = linoleic acid; ARA = arachidonic acid; EPA = eicosapentaenoic acid; DPA = docosapentaenoic acid; DHA = docosahexaenoic acid; SD = standard deviation; ND = non-detectable (ND defined as fatty acids detected with lower than 0.005% contribution to the overall fatty acid content).

**Table 7:** Fatty acid profiles of the FFAs present in TAC extracts (unsaponified samples) from all shrimp species, expressed as the mean for each FA (with the relevant standard deviation; SD); percentage composition of the FFAs in each extract assessed (n = 3).

Fatty Acid Emperical Name	Type of Fatty Acid (Carbon Atoms, Double Bonds, and Their Positions)	*L. vannamei*		*P. kerathurus*		*A. foliacea*		*P. longirostris*	
		Mean	SD	Mean	SD	Mean	SD	Mean	SD
Myristic	C14:0	0.3	0.0	1.1	0.0	0.6	0.0	0.3	0.0
Pentadecylic	C15:0	0.2	0.0	1.1	0.1	0.3	0.0	0.3	0.0
Palmitic	C16:0	9.1	0.3	16.4	0.3	14.8	0.2	5.0	0.6
Palmitoleic	C16:1 c9 (n7 MUFA)	1.3	0.0	5.7	0.0	6.3	0.1	6.5	0.5
Margaric	C17:0	0.5	0.0	3.2	0.1	0.8	0.0	0.5	0.0
Stearic	C18:0	6.6	0.3	12.2	0.7	5.9	0.1	3.2	0.5
OA	C18:1 c9 (n9 MUFA)	19.4	0.1	12.4	0.2	16.8	1.2	16.4	1.4
LA	C18:2 c9,12 (n6 PUFA)	31.0	0.1	1.7	0.0	3.1	0.0	1.0	0.1
ALA	C18:3 c9,12,15 (n3 PUFA)	1.6	0.0	0.8	0.0	0.4	0.0	0.3	0.1
Stearidonic	C18:4 c6,9,12,15 (n3 PUFA)	0.1	0.0	0.3	0.0	0.1	0.0	0.4	0.0
Nonadecylic	C19:0	0.0	0.0	0.2	0.0	0.3	0.0	0.0	0.0
Gadoleic	C20:1 c9 (n11 MUFA)	1.2	0.0	7.2	0.2	11.4	0.1	1.8	0.1
Dihomolinoleic	C20:2 c10,12 (n6 PUFA)	2.6	0.0	1.9	0.0	1.4	0.0	1.0	0.1
Dihomolinolenic	C20:3 c8,11,14 (n6 PUFA)	0.5	0.0	0.7	0.0	1.1	0.0	0.4	0.0
ARA	C20:4 c5,8,11,14 (n6 PUFA)	2.7	0.0	7.9	0.1	7.4	0.1	9.3	0.7
EPA	C20:5 c5,8,11,14,17 (n3 PUFA)	7.2	0.1	13.4	0.1	13.2	0.9	27.5 ^#^	2.2
Docosadienoic	C22:2 c13,16 (n6 PUFA)	0.0	0.0	1.7	0.0	0.3	0.0	0.4	0.0
Eranthic	C22:3 c5,13,16 (n6 PUFA)	0.0	0.0	0.4	0.0	0.0	0.0	0.1	0.0
Ardenic	C22:4 c7,10,13,16 (n6 PUFA)	0.1	0.0	1.6	0.0	0.4	0.0	1.1	0.1
DPA	C22:5 c7,10,13,16,19 (n3 PUFA)	0.5	0.0	1.2	0.0	1.7	0.0	3.5	0.3
DHA	C22:6 c4,7,10,13,16,19 (n3 PUFA)	15.1	0.1	8.9	0.1	13.7	0.9	20.9 ^#^	6.5
SFA		16.7	0.1	34.1	0.7	22.7	0.3	9.3	1.2
UFA		83.2 **	0.1	65.6 **	0.7	77.3 **	0.3	90.6 **	1.2
MUFA		21.9	0.1	25.3	0.4	34.5	1.3	24.7	1.9
PUFA		61.3 *	0.0	40.3 *	0.3	42.8 *	1.6	65.9 *	3.1
n3PUFA		24.5	0.1	24.5	0.2	29.1	1.6	52.6	4.0
n6PUFA		36.9	0.1	15.8	0.2	13.7	0.1	13.2	0.9
n6/n3		1.5	0.0	0.6	0.0	0.5	0.0	0.3 ^#^	0.0

** denotes statistically significant difference (*p* < 0.05) in UFAs compared with SFAs; * denotes statistically significant difference (*p* < 0.05) in PUFAs and compared with MUFAs; ^#^ denotes statistical significant difference between the values of EPA, DHA, n3 PUFAs, and n6–n3 ratio of *P. longirostris* compared with these values for other shrimp species. Abbreviations: n3 = omega-3; PUFA = polyunsaturated fatty acid; n6, omega-6; MUFA = monounsaturated fatty acid; SFA = saturated fatty acid; OA = oleic acid; ALA = alpha linolenic acid; LA = linoleic acid; ARA = arachidonic acid; EPA = eicosapentaenoic acid; DPA = docosapentaenoic acid; DHA = docosahexaenoic acid; SD = standard deviation; ND = non-detectable (ND defined as fatty acids detected with lower than 0.005% contribution to the overall fatty acid content).

**Table 8:** LC-MS analysis of representative polar lipid bioactives detected in TAC extracts from all shrimp species.

	PC (Molecular Species Detected) *			PE (Molecular Species Detected) *		
Shrimp Species	Elution Time (Min)	Mr (-CH2^−^)	PC Species	Elution Time (Min)	Mr-(H^−^)^−^	PE Specie
*L. vannamei*	8.0–8.5	800.6	PC O-40:8 (i.e., PC O-18:2/22:6 or PC O-20:4/20:4)	7.3–7.8	820.2	PE O-44:12 (i.e., PE O-22:6/22:6)
	9.0–9.5	842.5	PC 42:8 (i.e., PC 20:2/22:6)	8.3–8.8	734.0	PE 36:6 (i.e., PE 16:1/20:5 or PE 18:3/18:3)
	9.0–9.5	842.5	PC O-42:9;O (i.e., PC O-20:4/20:5;O)	8.0–8.5	800.6	PE O-42:8 (i.e., PE O-20:2/22:6)
	10.0–10.5	714.4	PC 32:2 ( i.e., PC 16:1/16:1)	10.0–10.5	698.4	PE O-34:3 (i.e., PE O-16:1/18:2 or PE O-16:0/18:3)
	10.0–10.5	812.4	PC 40:9 (i.e., PC 20:4/20:5 or PC 18:3/22:6)	10.0–10.5	714.4	PE 34:2 (i.e., PE 16:0/18:2 or PE 16:1/18:1)
	10.0–10.5	812.4	PC O-40:10;O (i.e., PC O-20:5/20:5;O)	10.0–10.5	714.4	PE O-34:3;O (i.e., PE O-16:1/18:2;O or PE O-16:0/18:3;O)
	10.0–10.5	828.4	PC 40:9;O (i.e., PC 20:4/20:5;O or PC 18:3/22:6;O)	10.0–10.5	744.4	PE O-38:8 (i.e., PE O-18:3/20:5)
				10.0–10.5	812.4	PE 42:9 (i.e., PE 20:4/22:5)
				10.0–10.5	812.4	PE O-42:10;O (i.e., PE O-20:4/22:6;O)
				10.0–10.5	828.4	PE 42:9;O (i.e., PE 20:4/22:5)
*P. kerathurus*	8.3–8.8	800.5	PC O-40:8 (i.e., PC O-20:4/20:4 or PC O-18:3/22:6)	8.3–8.5	800.5	PE O-42:8 (i.e., PE O-20:4/22:4)
	8.5–9.4	827.0	PC O-42:9 (i.e., PC O-20:5/22:4 or PC O-20:4/22:5)	8.5–9.4	827.0	PE O-44:9 (i.e., PE O-22:4/22:5)
	9.2–9.6	850.6	PC O-44:11 (i.e., PC O-22:5/22:6)	10.0–10.4	698.4	PE O-34:3 (i.e., PE O-16:1/18:3)
	10.0–10.4	714.4	PC 32:2 (i.e., PC 16:1/16:1)	10.0–10.4	714.4	PE 34:2 (i.e., PE 16:1/18:1 or PE 16:0/18:2 )
	10.0–10.4	744.4	PC 34:1 (i.e., PC 16:0/18:1 or PC 18:0/16:1)	10.0–10.4	714.4	PE O-34:3;O (i.e., PE O-16:0/18:3;O or PE O-16:1/18:3;O)
	10.0–10.4	744.4	PC O-34:2;O (i.e., PC O-16:1/18:1;O or PC O-16:0/18:2;O)	10.0–10.4	744.4	PE 36:1 (i.e., PE 18:0/18:1 or PE 16:0/20:1)
	10.0–10.4	828.4	PC 40:9;O (i.e., PC 20:4/20:5;O or PC 18:3/22:6;O)	10.0–10.4	744.4	PE O-36:2;O (i.e., PE O-18:1/18:1;O or PE O-16:0/20:2;O)
	10.3–10.8	850.6	PC O-44:11 (i.e., PC O-22:5/22:6)	10.0–10.4	828.4	PE 42:9;O (i.e., PE 20:4/22:5;O)
	10.3–10.8	792.7	PC 38:5 (i.e., PC 18:0/20:5 or PC 18:1/20:4)	10.4–10.9	792.7	PE 40:5 (i.e., PE 20:1/20:4 or PE 18:1/22:4)
	12.3–12.8	762.5	PC 36:6 (i.e., PC 16:1/20:5 or PC 18:3/18:3)	12.3–12.8	792.7	PE O-40:6;O (i.e., PE O-20:1/20:5;O or PE O-18:0/22:6;O)
				12.3–12.8	762.5	PE 38:6 (i.e., PE 18:1/20:5 or PE 16:0/22:6)
				12.3–12.8	762.5	PE O-38:7;O (i.e., PE O-16:1/22:6;O or PE O-18:2/20:5;O)
*A.* *foliacea*	8.4–9.6	777.0	PC O-38:6 (i.e., PC O-16:0/22:6 or PC O-18:1/20:5)	1.3–1.4	736.0	PE 36:5 (i.e., PE 16:0/20:5 or PE 16:1/20:4)
	8.2–8.4	826.6	PC O-42:9 (i.e., PC O-20:4/22:5 or PC O-20:3/22:6)	1.2–1.5	736.0	PE O-36:6;O (i.e., PE O-16:1/20:5;O)
	8.5–11.2	749.0	PC O-36:6 (i.e., PC O-16:1/20:5)	8.3–9.4	777.0	PE O-40:6 (i.e., PE O-20:1/20:5 or PE O-18:1/22:5)
	8.5–10.8	763.0	PC 36:6 (i.e., PC 16:1/20:5)	8.3–8.7	826.6	PE O-44:9 (i.e., PE O-20:4/22:5 or PE O-20:3/22:6)
	8.5–9.9	789.0	PC 36:1;O (i.e., PC 16:0/20:1;O or PC 18:0/18:1)	8.5–9.4	804.0	PE O-42:6 (i.e., PE O-20:1/22:5 or PE O-20:2/22:4)
	8.5–9.4	805.0	PC O-40:6 (i.e., PC O-20:1/20:5 or PC O-18:1/22:5)	8.5–9.7	748.0	PE O-38:6 (i.e., PE O-16:0/22:6 or PE O-18:1/20:5)
	8.5–9.9	879.0	PC 44:12;O (i.e., PC 22:6/22:6;O)	8.5–9.9	788.0	PE 38:1;O (i.e., PE 18:0/20:1;O)
	9.2–9.5	762.5	PC 34:0;O (i.e., PC 16:0/18:0;O or PC 17:0/17:0;O	9.2–9.6	762.5	PE 36:0;O (i.e., PE 18:0/18:0;O)
	9.6–10.0	838.6	PC 42:10 (i.e., PC 20:4/22:6)	9.6–11.2	824.0	PE O-44:10 (i.e., PE O-22:5/22:5)
	9.6–10.0	838.6	PC O-42:11;O (i.e. PC O-20:5/22:6;O)	10.4–10.8	792.8	PE 40:5 (i.e., PE 20:1/20:4 or PE 18:0/22:5)
	9.8–11.3	825.0	PC O-42:10 (i.e., PC O-20:4/22:6 or PC O-20:5/22:5)	10.6–12.1	777.0	PE O-40:6 (i.e., PE O-20:1/20:5 or PE O-18:1/22:5)
	10.4–10.8	792.8	PC 38:5 (i.e., PC 18:1/20:4 or PC 16:0/22:5)	10.6–12.1	818.8	PE 42:6 (i.e., PE 20:1/22:5 or PE 20:2/22:4)
	10.6–12.1	777.0	PC O-38:6 ( i.e., PC O-16:0/22:6 or PC O-18:1/20:5)	10.6–12.1	818.8	PE O-42:7;O (i.e., PE O-20:1/22:6;O or PE O-20:2/22:5;O)
	10.6–11.0	818.8	PC O-40:7;O (i.e., PC O-18:1/22:6;O or PC O-18:2/22:5;O)	11.1–11.4	816.6	PE 42:7 (i.e., PE 20:1/22:6 or PE 20:2/22:5)
	11.1–11.4	816.6	PC 40:7 (i.e., PC 18:1/22:6 or PC 18:2/22:5)	11.1–11.4	816.6	PE O-42:8;O (i.e., PE O-20:2/22:6;O or PE O-20:4/22:4;O)
	11.1–11.4	816.6	PC O-40:8;O (i.e., PC O-20:4/20:4;O or PC O-18:2/22:6;O)	11.1–11.4	805.0	PE O-42:6 (i.e., PE O-20:1/22:5 or PE O-20:2/22:4)
	11.2–12.1	851.0	PC O-44:11 (i.e., PC O-22:5/22:6)	11.1–11.4	763.0	PE O-38:7;O (i.e., PE O-16:1/22:6 or PE O-18:2/20:5)
	11.2–12.5	763.0	PC 36:6 (i.e., PC 16:1/20:5)	11.4–11.8	750.5	PE O-38:5 (i.e., PE O-18:1/20:4 or PE O-16:0/22:5)
	11.4–11.8	750.5	PC O-36:5 (i.e., PC O-16:0/20:5 or PC O-16:1/20:4)	11.4–11.8	778.6	PE O-40:5 (i.e., PE O-20:1/20:4 or PE O-18:0/22:5)
	11.4–11.8	778.6	PC O-38:5 (i.e., PC O-18:1/20:4 or PC O-16:0/22:5)	11.4–11.8	814.5	PE 42:8 (i.e., PE 20:2/22:6 or PE 20:4/22:4)
	11.4–11.8	814.5	PC 40:8 (i.e., PC 20:4/20:4 or PC 18:2/22:6)	11.4–11.8	814.5	PE O-42:9;O (i.e., PE O-20:4/22:5;O or PE O-20:3/22:6;O)
	11.4–11.8	838.6	PC 40:4;O (i.e., PC 20:1/20:3;O or PC 18:0/22:4;O)	11.6–12.5	763.0	PE 38:6 (i.e., PE 16:0/22:6 or PE 18:1/20:5)
*P. longirostris*	7.5–9.4	791.0	PC 38:6 (i.e., PC 16:0/22:6 or PC 18:1/20:5)	7.8–8.2	808.6	PE 40:5;O (i.e., PE 18:1/22:4;O or PE 20:1/20:4;O)
	7.8–8.2	808.6	PC 38:5;O (i.e., PC 18:1/20:4;O or PC 18:0/20:5;O)	8.2–8.4	736.5	PE 36:5 (i.e., PE 16:0/20:5 or PE16:1/20:4)
	7.8–8.2	864.6	PC 44:11 (i.e., PC 22:5/22:6)	8.2–8.4	802.6	PE O-42:7 (i.e., PE O-20:1/22:6)
	8.0–9.4	791.0	PC O-38:7;O (i.e., PC O-18:2/20:5;O or PC O-16:1/22:6;O)	8.3–9.4	791.0	PE O-40:7;O (i.e., PE O-18:1/22:6;O or PE O-18:2/22:5;O)
	8.0–8.4	864.0	PC O-44:12;O (i.e., PC O-22:6/22:6;O)	8.3–9.9	825.0	PE O-44:10 (i.e., PE O-22:5/22:5)
	8.1–8.3	802.6	PC O-40:7 (i.e., PC O-18:1/22:6)	8.5–9.9	791.0	PE 40:6 (i.e., PE 18:0/22:6 or PE18:1/22:5)
	8.3–8.7	862.6	PC 42:6;O (i.e., PC 20:1/22:5;O)	9.0–9.9	837.0	PE 42:5;O (i.e., PE 20:1/22:4;O)
	8.6–9.9	851.0	PC O-44:11 (i.e., PC O-22:5/22:6)	9.9–10.7	811.0	PE 40:4;O (i.e., PE 18:0/22:4;O)
	9.0–9.9	837.0	PC 40:5;O (i.e., PC 18:1/22:4;O or PC 20:1/20:4;O)	10.0–10.4	714.4	PE 34:2 (i.e., PE 16:1/18:1 or 16:0/18:2)
	9.0–9.4	865.0	PC 42:5;O (i.e., PC 20:1/22:4;O)	10.0–10.4	714.4	PE O-34:3;O (i.e., PE O-16:1/18:2;O)
	9.9–10.7	811.0	PC 38:4;O (i.e., PC 16:0/22:4;O or PC18:0/20:4;O)	10.0–10.4	744.4	PE 36:1 (i.e., PE 18:0/18:1)
	10.0–10.4	714.4	PC 32:2 (i.e., PC 16:1/16:1)	10.2–12.6	777.0	PE O-40:6 (i.e., PE O-18:0/22:6 or 18:1/22:5)
	10.0–10.4	744.4	PC 34:1 (i.e., PC 16:0/18:1 or PC 18:0/16:1)	10.4–12.6	763.0	PE 38:6 (i.e., PE 16:0/22:6 or PE 18:1/20:5)
	10.2–12.6	777.0	PC O-38:6 (i.e., PC O-16:0/22:6 or PC O-18:1/20:5)	10.6–12.6	749.0	PE O-38:6 (i.e., PE O-16:0/22:6 or PE O-18:1/20:5)
	10.2–10.6	824.6	PC O-42:10 (i.e., PC O-20:4/22:6 or PC O-20:5/22:5)	10.6–12.6	763.0	PE O-38:7;O (i.e., PE O-18:2/20:5;O or PE O-16:1/22:6;O)
	10.4–12.6	749.0	PC O-36:6 (i.e., PC O-16:1/20:5)	10.6–12.6	789.0	PE 40:7 (i.e., PE 18:1/22:6 or PE 18:2/22:5)
	10.6–12.6	763.0	PC 36:6 (i.e., PC 16:1/20:5)	12.3–12.8	722.5	PE O-36:5 (i.e., PE O-16:0/20:5 or PE O-16:1/20:4)
	10.8–12.6	789.0	PC 38:7 (i.e., PC 18:2/20:5)	12.3–12.8	788.5	PE O-40:8;O (i.e., PE O-20:4/20:4 or PE O-20:3/20:5)
	12.3–12.7	804.6	PC O-40:6 (i.e., PC O-18:0/22:6 or PC O-18:1/22:5)	12.3–12.8	804.6	PE O-42:6 (i.e., PE O-20:1/22:5)
	12.3–12.7	826.6	PC O-42:9 (i.e., PC O-20:4/22:5 or PC O-20:5/22:4)	12.3–12.8	826.6	PE O-44:9 (i.e., PE O-22:4/22:5)
	12.3–12.7	852.6	PC O-44:10 (i.e., PC O 22:5/22:5)			

* Abbreviations: PC = phosphatidylcholine; PE = phosphatidylethanolamine; TAC, total amphiphilic compounds.

## Data Availability

All data is contained within the article. For any further information concerning raw data (i.e., FT-IR Spectra, LC-MS chromatograms and Spectra) they can be provided by the Authors upon request.
